# The Impact of Glycosylation on the Functional Activity of CD133 and the Accuracy of Its Immunodetection

**DOI:** 10.3390/biology13060449

**Published:** 2024-06-18

**Authors:** Alisa Gisina, Konstantin Yarygin, Alexey Lupatov

**Affiliations:** Laboratory of Cell Biology, V. N. Orekhovich Institute of Biomedical Chemistry, 119121 Moscow, Russia

**Keywords:** CD133, prominin-1, N-glycosylation, immunodetection, antibodies

## Abstract

**Simple Summary:**

CD133 is one of the most relevant molecular markers of cancer stem cells, which are responsible for tumor relapse and progression. Although the role of CD133 in maintaining cancer cell stemness is not entirely clear, its involvement in a number of molecular mechanisms leading to a more malignant cellular phenotype has recently been demonstrated. Since CD133 is a glycoprotein, it is likely that the attached carbohydrates influence its function. In this review, we present and discuss the currently available data on CD133’s glycosylation and its impact on the functional activity of this molecule. Special attention is paid to the influence of the carbohydrates on the accuracy of CD133 detection using antibodies. This information will be useful for researchers involved in both basic CD133 research and that aiming at developing new methods for the prediction and control of tumor progression.

**Abstract:**

The membrane glycoprotein CD133 (prominin-1) is widely regarded as the main molecular marker of cancer stem cells, which are the most malignant cell subpopulation within the tumor, responsible for tumor growth and metastasis. For this reason, CD133 is considered a promising prognostic biomarker and molecular target for antitumor therapy. Under normal conditions, CD133 is present on the cell membrane in glycosylated form. However, in malignancies, altered glycosylation apparently leads to changes in the functional activity of CD133 and the availability of some of its epitopes for antibodies. This review focuses on CD133’s glycosylation in human cells and its impact on the function of this glycoprotein. The association of CD133 with proliferation, differentiation, apoptosis, autophagy, epithelial–mesenchymal transition, the organization of plasma membrane protrusions and extracellular trafficking is discussed. In this review, particular attention is paid to the influence of CD133’s glycosylation on its immunodetection. A list of commercially available and custom antibodies with their characteristics is provided. The available data indicate that the development of CD133-based biomedical technologies should include an assessment of CD133’s glycosylation in each tumor type.

## 1. Introduction

The ongoing interest in the CD133 molecule is due to its strong association with the phenotypes of stem and progenitor cells, including cancer stem cells, which are responsible for tumor relapses and metastasis [1,2]. In this regard, CD133 is often considered as a potential prognostic biomarker for various oncological diseases, as well as a promising target for cancer therapy [3,4]. The association of CD133 with cancer progression and poor prognosis was shown in meta-analyses devoted to such malignancies as breast cancer [5], colorectal cancer [6,7,8,9], gastric cancer [10,11], glioma [12,13,14], head and neck squamous cell carcinoma [15], hepatocellular carcinoma [16,17,18], non-small cell lung cancer [19,20,21], osteosarcoma [22], ovarian cancer [23,24] and pancreatic ductal adenocarcinoma [25]. Along with other reasons for which CD133 has still not found its place in clinical oncology (as reviewed in ref. [26]), the difference in CD133’s immunodetection patterns when using different antibodies is of great importance [27,28]. This phenomenon is often explained by the putative impact of glycosylation on the CD133 epitopes. Therefore, there may be an assumption that some antibody clones are able to exclusively recognize glycosylated CD133, while others recognize its non-glycosylated form.

Understanding the impact of glycosylation on CD133’s functional activity may be helpful in developing CD133-targeted anticancer therapies. Generally, glycosylation strongly affects a number of important processes related to the turnover of proteins, including protein folding, stability, the transport of proteins to the proper destination, protein solubility, etc. [29,30]. Moreover, changes in glycan structure can have dramatic effects on protein function. For example, the functional activity of proteins may depend on glycosylation at specific sites [31,32]. There is evidence that changes in the glycosylation of surface receptors affect their conformation and lead to impaired receptor binding with their external ligands or intracellular signaling molecules [33,34]. Aberrant glycosylation caused by mutations in genes that regulate the post-translational modification of proteins is associated with a number of serious diseases [35,36]. 

This information is expected to be important when developing new biomedical technologies. In this review, we consider the latest data concerning the effect of CD133’s glycosylation on its functional activity. This could be useful when developing new antitumor therapies using CD133 as a target molecule to eliminate cancer stem cells. In addition, this review provides data regarding the influence of glycosylation on CD133’s immunodetection and includes a list of commercial and custom antibodies, along with the available information about their ability to bind CD133 with different glycosylation statuses. This information is intended to help in selecting the appropriate antibodies for various applications. 

## 2. Role of CD133 in Cells

Human CD133 was originally described as a molecule expressed exclusively on hematopoietic stem and progenitor cells [37]. However, later, it was found to be expressed across a much wider range of cell types [27,38,39]. CD133 expression starts in the embryo and persists throughout life in a wide range of normal and cancer cell types [40]. CD133 is expressed on quiescent and actively proliferating stem cells that contribute to the growth and maintenance of normal and tumor tissue and sometimes on differentiated epithelial cells prone to dedifferentiation [41]. However, it remains unclear whether CD133 is essential for the maintenance of the stem cell phenotype [42] or is just a bystander involved in the physiological processes that take place in stem cells but are unrelated to the stemness state itself [43,44]. There are data that contradict the concept of CD133’s involvement in cell stemness. For example, in the human embryonic stem cell line WA26, the knockout of CD133 significantly reduced cells’ proliferation but did not affect their pluripotency [45]. Moreover, all described CD133-knockout mouse models were viable and had a limited range of developmental defects [46,47,48,49]. In addition, the possibility of tumor formation from a cell population depleted of CD133-expressing cells was shown [50].

The role of CD133 in the photoreceptor cells of various animals and humans has been studied in detail. In humans and laboratory rodents, it is expressed in the disk membranes of rods and cones throughout life, starting from embryogenesis [51]. Mutations of the *PROM1* gene encoding CD133 lead to visual impairment [52], and the knockout of the *PROM1* gene in experimental animals leads to complete blindness [46,53]. It is suggested that the role of CD133 in visual cells is a consequence of its involvement in the formation of the cell membrane architecture, since it has been observed that, in other types of cells, it is concentrated predominantly in the protrusions of the cell membrane—microvilli and cilia [54]. There is also information about its relationship with the elements of the cytoskeleton [55,56]. CD133 is not homogenously distributed throughout the cell membrane but is concentrated within the cholesterol-containing lipid rafts [54,57]. A remarkable biological feature of CD133 is its release in several bodily fluids in association with membrane particles, referred to as “prominosomes” [58,59,60,61]. The presence of prominosomes has been demonstrated in biofluids, including urine, saliva, and cerebrospinal fluid [27,38,61,62,63,64,65]. Presumably, CD133 is involved in intracellular communication through the release of prominosomes and also through CD133 transport via tunneling nanotubes [66,67]. Recently, data have been published on the involvement of CD133 in the initiation of autophagy during nutrient deprivation in the cell microenvironment [68]. It is likely that the involvement of CD133 in the endocytic–exocytic pathway is associated specifically with the process of autophagy and the recycling of damaged/unnecessary organelles to avoid apoptosis. In particular, the process of the initiation of autophagy, triggered by CD133, is accompanied by a reduction in cellular microvilli, which may be a manifestation of the process of cellular dedifferentiation [69]. Recently, it has been demonstrated that CD133 is also involved in adult liver regeneration after injury [70,71,72]. CD133 appears to be involved in several signaling pathways, specifically PI3K/Akt [73,74], Src/FAK [75,76], Wnt/β-catenin [77,78,79], TGF-β/Smad [80] and MAPK/ERK [81,82,83] (as reviewed in ref. [2]). This may explain the broad action of CD133 in many cellular processes, including cell proliferation [84,85], apoptosis [86,87] and epithelial–mesenchymal transition [88,89].

## 3. CD133 Glycosylation

**Figure 1:**
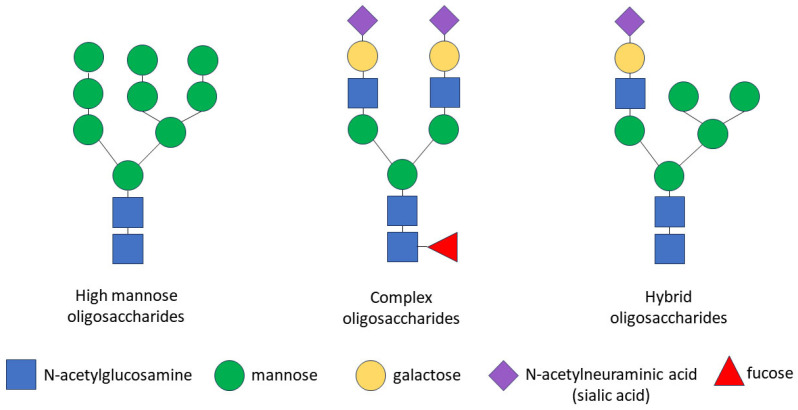
Schematic representation of N-glycan structures. There are three types of N-linked glycans: (1) high-mannose oligosaccharides, which contain many mannose residues; (2) complex oligosaccharides, which contain various types of sugars, including mannose, N-acetylglucosamine, galactose, N-acetylneuraminic acid (sialic acid) and fucose; (3) hybrid oligosaccharides, which contain branches of both high-mannose and complex oligosaccharides. Symbols are used according to the Symbol Nomenclature for Graphical Representation of Glycans [91].

The glycosylation of any protein is a co-translational and/or post-translational modification through the covalent attachment of an oligosaccharide to a protein molecule. This process occurs as a result of consecutively ordered enzymatic reactions in the endoplasmic reticulum (ER) and the Golgi apparatus [90]. Glycopeptide bonds can be categorized into specific groups based on the nature of the bond between the sugar and the peptide, including N-, O- and C-linked glycosylation, glypiation and phosphoglycosylation. Proteins may not be limited to a specific type of glycosylation and may be glycosylated at multiple sites via different glycosidic linkages [91]. Specifically, the CD133 glycoprotein carries only N-glycosidic linkages [92,93]. The N-linked glycosylation of proteins occurs in the ER and consists of three stages: the formation of a lipid-linked oligosaccharide precursor in the ER; the transfer of the oligosaccharide precursor to the nascent polypeptide chain with the concomitant cleavage of glucose residues; and the processing of the oligosaccharide chain to form a glycan core consisting of N-acetylglucosamine and mannose [35,94]. Some glycoproteins containing high-mannose N-glycans are transported through the Golgi apparatus to the plasma membrane without further processing, but most N-glycans are further modified in the Golgi apparatus, where glycan processing combines trimming and the addition of sugars, resulting in the diversification of the glycans within individual glycoproteins. In the Golgi apparatus, specific enzymes are segregated within different cisterns, allowing for a stepwise process. The final glycan structure can be categorized into three groups: high-mannose oligosaccharides, which contain many mannose residues; complex oligosaccharides, which contain many types of sugars, including mannose, N-acetylglucosamine, galactose, N-acetylneuraminic acid (sialic acid) and fucose; and hybrid oligosaccharides, which contain branches of both high-mannose and complex oligosaccharides (Figure 1). As a result of transformations in the Golgi apparatus, some glycosylation sites in the protein can be occupied by complex N-glycans and other sites by high-mannose or hybrid N-glycans [91,95]. 

**Figure 2:**
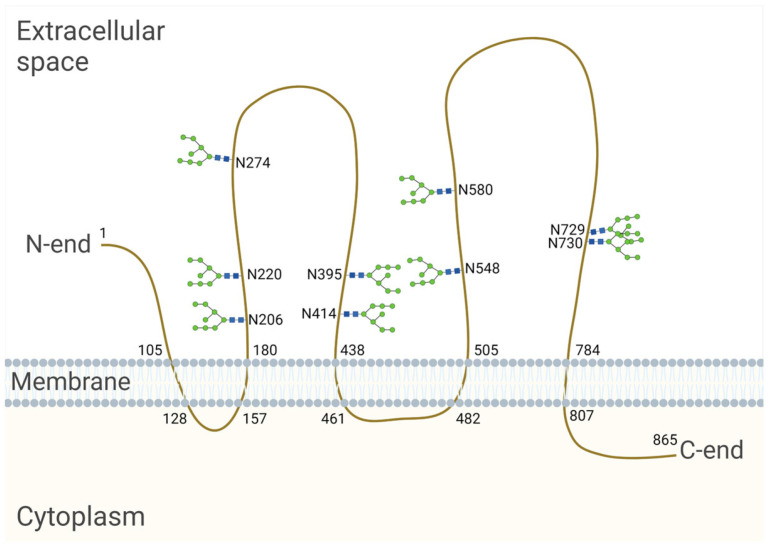
Model of the human CD133 glycoprotein placed in the cell membrane. The longest splice variant of human CD133 (CD133s2) consists of 865 amino acid residues [100] and has nine sites of N-glycosylation [93]. The numbers on the figure indicate the serial numbers of the amino acid residues in a polypeptide; N is a symbol denoting asparagine. The model is based on the sequence analysis and hydrophobicity analysis of the protein sequence [92], bioinformatic secondary structure prediction [101] and the site-specific characterization of N-glycosylation by mass spectrometry [93]. Created with BioRender.com (accessed on 3 March 2024).

The CD133-encoding gene, named *PROM1*, has six different promoters that are regulated in a tissue-dependent manner, leading to the expression of alternative CD133 splice variants [96]. The longest splice variant of the human CD133 polypeptide (CD133s2) consists of 865 amino acids [92]. When located on the cell surface, it spans the membrane five times and forms an extracellular N-terminus, two large glycosylated extracellular loops and an intracellular C-terminus [92] (Figure 2). Nascent CD133, which is located in the ER and/or early Golgi compartments, is an N-linked high-mannose molecule weighing approximately 105 kDa [97]. After processing in the Golgi apparatus, the molecular weight of CD133 increases by ≈20–30 kDa, and, as a result, the molecular weight of fully glycosylated CD133 is 120–130 kDa [92,97,98]. The molecular weight of the deglycosylated CD133 polypeptide is 92–97 kDa [92,97]. An individual splice variant can presumably exist in several different glycoforms with variable molecular weights depending on the length of the splice variants and their level of glycosylation [99].

Using HEK293T cells, Liu et al. revealed that human CD133 contains nine potential N-glycosylation sites on two large extracellular loops (Asn206, Asn220, Asn274, Asn395, Asn414, Asn548, Asn580, Asn729, Asn730) [93] (Figure 2). It is known that the transfer of a glycan to a nascent protein during N-glycosylation occurs co-translationally when it is translated and transported to the ER. Glycans are covalently linked to the nitrogen atom at asparagine (Asn) residues, which are present as part of the consensus sequence Asn—X—Ser/Thr, where X is any amino acid other than proline (Pro). However, it is important to note that not all Asn residues with a consensus sequence are glycosylated. Protein synthesis from the N- to the C-terminus results in the transport of the nascent polypeptide into the ER in the same orientation, and protein folding occurs shortly after the polypeptide enters the ER. As protein folding progresses, it becomes more difficult for specific enzymes to reach a glycosylation site for glycan transfer [94]. In HEK293 cells, it was found that replacing Asn with Gln at individual CD133 N-glycosylation sites had no effect on the expression level or membrane localization. At the same time, the mutation of all N-glycosylation sites affected the surface localization of CD133 and led to the retention of the molecule in the ER, as determined by co-localization with calnexin [93,102]. Calnexin is an ER lectin that specifically interacts with the nascent glycans attached to polypeptides and controls the proper folding and assembly of glycoproteins upon N-glycosylation. It is likely that a CD133 mutant with mutations at all N-glycosylation sites is unable to bind to calnexin, resulting in improper protein folding and a failure to pass quality control in the ER [102].

## 4. The Role of Glycosylation in the Functional Activity of CD133

When comparing normal and tumor tissue, it was revealed that the expression of CD133 in the vast majority of cases was significantly higher in the latter [42,103,104,105,106,107,108]. In addition, there was evidence that CD133’s expression was higher in samples from patients with metastases and at advanced stages of cancer [10,11,16,22,24,25]. These data could be explained by the involvement of CD133 in cell proliferation and anti-apoptosis. In fact, it was revealed that the intracellular interaction of CD133 with HDAC6 leads to the stabilization of β-catenin and subsequent activation of the Wnt/β-catenin signaling pathway, affecting cell proliferation [77]. Moreover, CD133 was found to be involved in the activation of the PI3K/Akt signaling pathway through direct intracellular interaction with the PI3K p85 subunit [73]. Specifically, the phosphorylation of the tyrosine 828 residue in CD133’s C-terminal cytoplasmic domain mediates this interaction. The activation of Akt then leads to the increased activity of anti-apoptotic factors [109]. Liu et al. found that a mutation at the Asn548 N-glycosylation site significantly reduced the viability and colony formation of hepatoma cells [93]. To examine the mechanism by which the Asn548 mutation reduced the ability of CD133 to promote cell growth, the authors investigated its effect on CD133’s interaction with β-catenin and PI3K. It was revealed that the Asn548 mutation significantly reduced the binding of CD133 to β-catenin. Moreover, the Asn548 mutation reduced the β-catenin protein level and β-catenin signaling. However, a mutation at the Asn548 N-glycosylation site did not change Y828’s phosphorylation in CD133. Thus, a correlation between the functional role of Asn548 glycosylation and the binding of the phosphorylated residue Y828 to the p85 regulatory subunit of PI3K in hepatocellular cancer lines was excluded [93].

In a study of neural stem cells and glioma-initiating cells, it was found that the terminal N-glycan groups of CD133 were sialylated [110]. This terminal modification contributed to protein stability, allowing CD133 to escape lysosomal degradation. The N-terminal glycan groups of CD133 were sialylated via an α2,3-linkage, and its desialylation by the enzyme neuraminidase led to instability and degradation through a lysosomal-dependent pathway [110]. In general, CD133’s sialylation may be important in cancer progression, as changes in this post-translational modification play a role in invasion and metastasis [111,112]. Glycoprotein sialylation can mediate cell–cell interactions, extracellular matrix interactions, ligand–receptor interactions and intracellular downstream signaling in several biological processes [113]. In a study by Sakaue et al., CD133’s expression was analyzed in exosomes extracted from the ascites of patients with inoperable pancreatic cancer [114]. A lectin microarray analysis identified the glycosylation of CD133 by sialic acids as a major glycosylation type among various other glycosylation types of exosomal CD133 [114]. 

The enzymes involved in the N-glycosylation of proteins include various glycosyltransferases and glycosidases—specifically, α-mannosidase (MAN) families I and II, N-glycan branching β-N-acetylglucosaminyltransferases (MGAT), β1,3-galactosyltransferases (b3GalT), β1,4-galactosyltransferases (b4GalT), α2,3-sialyltransferases (a3SAT), α2,6-sialyltransferases (α6SAT) and α1,2-/α1,3-/α1,4-/α1,6-fucosyltransferases (FucT). In HEK293 cells, as a result of genetic screening, the significant role of several genes in the processing of complex N-glycans in CD133+ cells was discovered—in particular, *MGAT3*, *MGAT4B*, *MGAT4C* and *MGAT5* [102]. The effect of complex CD133 N-glycan processing was also investigated by focusing on MGAT4C, which is a glycosyltransferase involved in catalyzing GlcNAc β1–4’s binding to the core mannose residues of N-glycans for tri- or tetraantennary N-glycan structures. MGAT4C was found to directly process complex CD133 N-glycans [102]. When comparing the glycan profiles of CD133+ and CD133− cord blood-derived cells, it was found that CD133+ cells had increased expression of MGAT2 and decreased expression of MGAT4. The overexpression of ST3GAL6 in CD133+ cells was also detected [115].

It has been demonstrated that, in glioma stem cells, the lower expression of mannosyl-oligosaccharide 1,2-α-mannosidase IA (MAN1A1) leads to the formation of a high-mannose type N-glycan on the CD133 molecule. The C-terminal domain of CD133 was also found to interact with DNA methyltransferase 1 (DNMT1), and the glycosylation status of CD133 influenced this interaction. Specifically, the high-mannose type of glycosylation was required for this interaction [116]. It was found that high-mannose CD133 maintained the glioma stem cells in a slow-cycling state by blocking the nuclear translocation of DNMT1. The activation of p21 and p27 through the CD133–DNMT1 interaction maintained the slow-cycling state of the glioma stem cells and stimulated their resistance to chemotherapy and tumorigenesis. In contrast, inducing the formation of complex N-glycans in CD133 through the ectopic expression of MAN1A stimulated the nuclear translocation of DNMT1. The blocking of the CD133–DNMT1 interaction or MAN1A1 overexpression inhibited the tumorigenesis of glioma stem cells and increased their sensitivity to temozolomide. Thus, the essential role of high-mannose N-glycans in the functioning of CD133 was shown [116].

α1,2-Mannosylated CD133 has been found to be a functional marker of intrahepatic cholangiocarcinoma-initiating cells [117]. The frequency of sphere formation by CD133+α-1,2-Man+ cells was higher than that by CD133− cells or CD133+α-1,2-Man− cells. The levels of stemness genes, including *SOX4*, *NANOG* and *POU5F1* (Oct-4), were high in CD133+α-1,2-Man+ cells. In addition, CD133+α-1,2-Man+ tumor cells were highly tumorigenic in immunodeficient mice. It was found that the expression level of the α1,2-mannosidase (MAN1C1) mRNA and protein was significantly lower in CD133+α1,2-Man+ cells. Lower expression of MAN1A1 has been shown to result in the formation of high-mannose N-glycans in CD133. However, the overexpression of MAN1C1 did not reduce the level of CD133 or affect the cell surface localization of CD133. It was found that the MAN1C1 enzyme levels were higher in cells with the membrane distribution of CD133, and α1,2-mannosylation promoted the cytoplasmic distribution of CD133. It was found that α1,2-mannosylation enhanced the self-renewal ability of cells, initiating intrahepatic cholangiocarcinoma by improving autophagy. α1,2-Mannosylation stimulated the endosomal distribution of CD133 and the interaction of endosomal CD133 with FIP200. The interaction between CD133 and FIP200 increased the stability of FIP200, a ULK-interacting protein required for autophagosome formation in mammalian cells. One potential explanation for this phenomenon is that the high-mannose CD133 accumulates in early endosomes due to a lack of mono-ubiquitination [117].

It was also found that in hypoxia, the stability of CD133 is regulated by glycosyltransferase 8 domain containing 1 (GLT8D1), a transmembrane glycosyltransferase that is highly expressed during hypoxia under the influence of the HIF1α transcription factor. GLT8D1 co-localizes with CD133 in the microvilli of glioma stem cells, where it glycosylates CD133, preventing its degradation through the endosomal–lysosomal pathway [118]. A GLT8D1 mutant deficient in glycosyltransferase activity could partially impede CD133 degradation, suggesting that even the physical association of GLT8D1 with CD133, independent of its activity, reduces CD133’s degradation. This indicates that both the enzymatic and anchoring roles of GLT8D1 are critical in inhibiting CD133’s degradation through the endosomal–lysosomal pathway. GLT8D1 interacts with the first extracellular domain of CD133 and prevents its lysosomal degradation through N-glycosylation and protein–protein interactions. As a result, the levels of CD133 and β-catenin increase and they stimulate Wnt/β-catenin signaling [118].

There is information about the hormonal regulation of CD133’s glycosylation [119]. The influence of maternal ovarian hormones on the expression and glycosylation of CD133 in uterine epithelial cells was found. Specifically, estrogen stimulated CD133 production, whereas progesterone altered the process of CD133’s glycosylation and stimulated its concentration at the apical region in uterine epithelial cells. These effects could be associated with the preparation of uterine epithelial cells for the implantation of the blastocyst through plasma membrane transformation during early pregnancy [119].

It is important to note that it is currently unknown to what extent differential glycosylation is specific to CD133, and it is not a general pattern for all membrane glycoproteins associated with a cellular state.

## 5. The Effect of CD133’s Glycosylation on Its Immunodetection

The results of CD133 immunodetection in different studies may vary depending on the sample preparation method. One of the main reasons for this is the usage of different anti-CD133 antibody clones [27,28,120]. There are antibodies to various parts of the CD133 molecule, including the extracellular N-terminus, intracellular C-terminus and extracellular loops. However, the detected patterns of CD133 expression in tissue may vary not only with the antibodies to the different parts of CD133, but even when using antibodies to the same part of the molecule. For example, the detection patterns of AC133 and W6B3C1 antibody clones differed in immunohistochemical studies [28]. Differences in expression patterns were also observed when using AC133 and AC141 antibody clones in flow cytometry [120]. Monoclonal antibody 80B258 revealed the widespread expression of CD133 in healthy adult human tissue, while no CD133 was detected in this tissue using antibody clone AC133 [27]. It is known that the binding sites for antibody clones AC133, AC141, W6B3C1 and 80B258 are located on the extracellular loops of CD133 [27,121,122]. It has been suggested that the glycosylation of CD133 may influence the conformation of the epitope recognized by a particular antibody clone. Moreover, there have been observations that the differential glycosylation status of CD133 is associated with cell differentiation [97,123]. The largest number of studies in this direction have been devoted to the nuances of recognition of the AC133 epitope, which is considered as the most reliable marker of normal progenitor cells and cancer stem cells [102,121,123]. It is worth noting that antibodies to CD133 are often suitable for use both when the native structure of the protein is preserved (flow cytometry, immunofluorescence) and when the protein is in a denatured state (Western blotting). This indicates the good spatial accessibility of the corresponding epitopes recognized by these antibodies. However, in some cases, special antibody clones could detect CD133 only in fixed cells. For example, under native conditions, 80B258, in contrast to AC133, did not recognize its matching epitope located on the second extracellular domain of human CD133 (amino acids Gly240–Ser388). At the same time, recognition could be made possible via the treatment of fixed cells with a low concentration of SDS [27]. However, the ability to bind exclusively glycosylated or exclusively non-glycosylated CD133 has not been demonstrated for either type of antibody. In Table 1 and Table 2, the application characteristics of commercial and custom antibodies against human CD133 are compiled. It should be noted that some of these antibodies react with CD133 in the cells of other species, e.g., mouse or rat.

It has been suggested that changes in the degree of glycosylation of the extracellular loops may explain the difference in the recognition of surface CD133 that is observed during cell differentiation [123,133] or hypoxia [134]. Such assumptions were prompted by the observation that when the level of detectable membrane CD133 changed, the expression level of the total CD133 mRNA and total protein did not change [121]. Changes in epitope recognition could theoretically be caused by either increases or decreases in the degree of glycosylation or a varying glycan composition. In particular, Hemmoranta et al. observed differences in the glycan structures of cord blood-derived CD133+ and CD133− cells [115]. The difference in neutral N-glycans’ profiles between CD133+ and CD133− cells was 9%. The difference in the profiles of sialylated N-glycans was 17%. Higher levels of high-mannose-type N-glycans, biantennary complex-type N-glycans with core composition H5N4 (five hexoses and four N-acetylhexosamines) and sialylated monoantennary N-glycans were detected in CD133+ cells, while, in CD133− cells, more complex-type N-glycans with core composition H6N5 (six hexoses and five N-acetylhexosamines) or larger, sialylated hybrid-type N-glycans and low-mannose-type N-glycans were found [115].

Taïeb et al. found that the extracellular N-terminal domain of the CD133 protein could undergo conformational modifications that can mask epitopes after the addition of membrane gangliosides [131]. The authors generated antipeptide antibodies against the N-terminal epitope of CD133 belonging to the ganglioside-binding domain. It is important to note that, unlike the extracellular loops, the N-terminal domain is non-glycosylated. Surprisingly, cell labeling with these antibodies was inhibited by gangliosides GM1 and GD3. However, ganglioside GT1b did not have such an effect. CD133 immunostaining was reduced to undetectable levels in post-confluent cultures, possibly through ganglioside-mediated epitope masking, since the staining was partially recovered after the chemical disruption of the lipid rafts. The authors suggested that the lipids surrounding the CD133 protein on the membrane may influence the accessibility of its epitopes to various antibodies [131].

The hypothesis that CD133 undergoes complex N-glycosylation and that this complexity is required for the expression of the specific AC133 epitope was explored in HEK293 cells [102]. To do this, a series of glycosidase treatments were performed using purified affinity-tagged CD133 to characterize each of the CD133 bands observed on the Western blot. Purified CD133 was first treated with PNGase F, an endoglycosidase that cleaves the GlcNAc bound to asparagine residues regardless of the N-glycan type. The treatment of CD133 with PNGase F caused the two parent bands to fuse together and shift to a molecular mass of approximately 100 kDa, which corresponded to the mass of the nascent CD133 protein. These changes in the molecular weight of the CD133-specific bands suggest that both bands represent CD133 bound to different types of N-glycans. In parallel, the authors treated purified CD133 with endoglycosidase Endo Hf, which is used in experiments to determine whether glycoproteins contain high-mannose or complex oligosaccharides, since any complex glycoproteins are resistant to glycan removal by endoglycosidase Endo Hf. In this case, only the lower CD133 band shifted to approximately 100 kDa, suggesting that this glycoform of CD133 is not complex. It is known that, without α-mannosidase II activity, the resulting outcome is the induction of hybrid N-glycans, but not complex ones. The pretreatment of cells with swainsonine, which is an inhibitor of α-mannosidase II and complex N-glycan biosynthesis, resulted in the inability of CD133 to undergo complex N-glycan processing and it was therefore sensitized to degradation by the Endo Hf enzyme. However, pretreatment with swainsonine led to a decrease in the molecular weight of CD133, but not its expression level. However, there was a significant decrease in the cell surface expression of the AC133 epitope, as determined by cytometry. This suggests that the complex N-glycosylation of CD133 contributes to the detection of the AC133 epitope on the cell surface. However, the inhibition of complex N-glycan processing in CD133 did not completely remove the AC133 epitope from the cell surface, suggesting that it is an important but not essential factor. Thus, it can be hypothesized that CD133 glycoforms created by the N-glycosylation of specific sites on CD133 and CD133 N-glycan processing lead to different degrees of cell surface recognition by AC133 antibody clones [102]. Another group of researchers also concluded that secreted CD133 has complex-type N-glycosylation and is modified by the N-glycan with a β1,6-linked terminal GlcNAc residue [135]. However, they found that the inhibition of the complex-type N-glycosylation of CD133 by swainsonine did not affect the membrane localization of CD133, but it significantly reduced the secretion of CD133 and stimulated its accumulation in early endosomes. Their results demonstrate the glycosylation-dependent mechanism of CD133 secretion [135].

## 6. Conclusions and Future Perspectives

It is now well known that the glycosylation of CD133 stabilizes this glycoprotein on the cell membrane, prevents CD133’s degradation through the endosomal–lysosomal pathway and activates cell autophagy. In addition, the role of CD133 glycosylation in the tumorigenicity and resistance to chemotherapy of CD133-positive tumor cells has been revealed. Obviously, CD133 glycosylation influences the degree of tumor cell malignancy. In this regard, the relationship between certain aberrations occurring in the tumor cell and the disruption of CD133 glycosylation must be explored in detail. Recent research has shown that specific patterns in glycan structures are associated with stemness and epithelial-to-mesenchymal transition [136], constituting essential features of cancer stem cells. If so, CD133 glycosylation may be a promising target for therapy.

The use of different clones of antibodies against CD133 can lead to differences in tissue staining patterns. Partly, this may be due to the detection of alternative splice variants, either tissue-specific or appearing in tumor cells [137]. However, the absence of binding to extracellular loops is more likely to indicate a change in glycosylation. For example, altered glycosylation explains the change in staining with certain antibodies during cell differentiation [97,123]. However, there is still no clear answer regarding which antibody clones recognize only glycosylated and which recognize non-glycosylated CD133 proteins. For some antibodies, the ability to bind both glycosylated and non-glycosylated CD133 proteins has been shown by Western blotting, which suggests the linearity of the recognized epitope. However, for many clones, there is no information about the type of epitope that they recognize, or such information is incomplete. 

To translate CD133 into a prognostic biomarker in the clinic, the analytical procedure must be standardized and quantification carried out with high accuracy. Unfortunately, the insufficient characterization of the available antibodies, the lack of a reliable 3D model of CD133 and the unpredictable glycosylation status in clinical tumor samples make this task challenging. However, sharing several clones or obtaining a new antibody with the specified properties could improve the accuracy in assessing the CD133 expression in the clinical setup. Further studies of the influence of the glycosylation status on CD133’s function and the state of CD133-positive cells are necessary to fully understand its role in the maintenance of stem and progenitor cells in normal and tumor tissue. This insight will provide new strategies for the detection and elimination of cancer stem cells in cancer patients. 

Future perspectives lie not only in the field of immunodetection. A promising method for CD133 detection is the use of nucleic acid aptamers. They are not only able to penetrate into the solid tumor better than antibodies but are also internalized in the cell by receptor-mediated endocytosis [138]. Aptamers can be used as a component in sensor detection systems [139] and in vectors for drug delivery to cancer stem cells [140,141,142,143,144]. In addition to aptamers, peptides capable of specific binding to CD133 can be selected using phage display peptide libraries and the systematic evolution of ligands by exponential enrichment [145]. These peptides can be used to develop CD133-targeted theranostics [146,147,148].

Another promising approach is to use mass spectrometry techniques to detect CD133. Since CD133 is involved in the formation of cell membrane protrusions, it is abundant in extracellular vesicles, including those formed by tumor cells [149]. Targeted quantitative proteomics, such as SRM analysis, can be applied to assess CD133 in exosomes and other vesicles from patient’s biofluids. A similar approach has been successful in detecting the high expression of CD133 in the exosomes of patients with autosomal dominant polycystic kidney disease [150]. The ability to assess CD133 expression in samples such as blood plasma greatly expands the potential for clinical use. In addition, targeted mass spectrometry allows us to evaluate CD133 regardless of its glycosylation status and even create a barcode to identify specific patterns of glycosylation. 

Among the cell-based approaches, chimeric antigen receptor T cells (CAR-T cells) targeting CD133 are the best choice for the elimination of cancer stem cells [151]. CAR-T cells must recognize the native CD133 antigen on a living cancer cell, and the antigen’s glycosylation can have a dramatic effect on the efficiency of the antigen’s binding to the receptor. For this reason, the design of a chimeric receptor should be based on data on the glycosylation patterns of the expected tumor target. Differences in the CD133 glycosylation status between normal and tumor cells may help to reduce the toxicity of CAR-T cell therapy. 

Thus, the further in-depth study of CD133’s glycosylation, and especially a comparison of its glycosylation in normal and various tumor cells, along with the detailed characterization of CD133 epitopes, should significantly improve the prognostic methods and the efficiency of targeted therapy for cancer.

## Figures and Tables

**Table 1:** Commercially available anti-human CD133 antibodies *.

Clone	Manufacturer	Host/ Clonality	Immunogen	Recognized Epitope	Application	Recognition of Glycosylated CD133	Recognition of Non-Glycosylated CD133
REA753	Miltenyi Biotec (Bergisch Gladbach, North Rhine-Westphalia, Germany)	HCL/m recombinant	No data	Epitope 1 on extracellular loops (CD133/1)	FC, MICS, IF, IHC, MC	No data	Yes M = 95 kDa
REA816	Miltenyi Biotec	HCL/m recombinant	No data	Epitope 2 on extracellular loops (CD133/2)	FC, MICS, IF, IHC, MC	Yes M = 117 kDa	No data
REA820	Miltenyi Biotec	HCL/m recombinant	No data	CD133/2	FC, MICS, IF, IHC	Yes M = 117 kDa	No data
REAL233	Miltenyi Biotec	CL/m recombinant	No data	CD133/1	FC, MICS, IF, IHC	No data	No data
AC141	Miltenyi Biotec	M/m	WERI-Rb-1 retinoblastoma cell line [37]	CD133/2	FC, MICS, IF, IHC	Yes M = 117 kDa	Yes [121]
293C3	Miltenyi Biotec; Abcam	M/m	Full-length human CD133	CD133/2	FC, IF, IHC, MICS	Yes M = 117 kDa	Yes [121]
AC133	Miltenyi Biotec	M/m	Hematopoietic stem cells [37]	CD133/1	FC, MICS, IF, IHC, ICC, MC, WB	Yes M = 117 kDa [92]	Yes [121] M = 97 kDa [92]
W6B3C1	Miltenyi Biotec; Abcam; Biolegend	M/m	WERI-RB-1 retinoblastoma cell line	CD133/1	WB, FC	Yes [121] M = 120 kDa	Yes M = 95 kDa [121]
EPR16508	Abcam (Cambridge, UK)	R/m	Recombinant fragment within human CD133	No data	WB, IHC-P	Yes M = 110 kDa	No data
EPR20980-45	Abcam	R/m	Recombinant fragment within human CD133	No data	WB, IHC-P	Yes M = 110–120 kDa	No data
RM1002	Abcam	R/m	Recombinant fragment within human CD133	No data	WB, IHC-P, FC, IP	Yes M = 120 kDa	No data
EPR20980-104	Abcam	R/m	Recombinant fragment within human CD133	No data	WB, IHC-P, FC, IP	Yes M = 110–120 kDa	No data
ab19898	Abcam	R/p	Synthetic peptide corresponding to human CD133 (C-terminal)	C-terminus	ICC, ICC/IF, FC, IHC-P, IHC-Fr, IP, WB	Yes M = 120 kDa	Yes M = 97 kDa
CMab-43	Abcam	M/m	LN229 glioblastoma cells expressing full-length CD133 [124]	No data	IHC-P, WB, FC [124]	Yes [124] M = 128 kDa	Yes [124] M = 97 kDa
Clone 7	Biolegend (San Diego, CA, USA)	M/m	Recombinant partial human CD133 protein—amino acid residues 180–380 and 612–765	Extracellular loops	FC, IHC-P, WB, IF	Yes [125] M = 130 kDa	Yes [125] M = 97 kDa
S16015F	Biolegend	M/m	Human CD133 transfectants	No data	FC	No data	No data
S16016B	Biolegend	M/m	Human CD133 transfectants	No data	FC	No data	No data
S16016E	Biolegend	M/m	Human CD133 transfectants	No data	FC	No data	No data
E-11	Santa Cruz (Santa Cruz, CA, USA)	M/m	No data	Between amino acids 841 and 865 at the C-terminus	WB, IHC-P, IP, IF, ELISA, FC	Yes M = 110–120 kDa	No data
TMP4	Thermo Fischer (Waltham, MA, USA)	M/m	No data	CD133/1	FC	No data	No data
EMK08	Thermo Fischer	M/m	No data	CD133/1	FC, ICC, IF	No data	No data
2F8C5	Thermo Fischer	M/m	Purified recombinant fragment of human CD133 (amino acids 20–108) expressed in E. coli	No data	ICC, IF, IHC-P, WB	Yes M = 130 kDa	No data
5E3	Thermo Fischer	M/m	Recombinant full-length human CD133	No data	WB	Yes M = 133 kDa	No data
BLR093G	Thermo Fischer	R/m recombinant	Amino acids between 350 and 400	No data	WB, IHC-P, ICC, IF	Yes M = 110–130 kDa	No data
D2V8Q	Cell Signaling Technology (Danvers, MA, USA)	R/m recombinant	Recombinant protein specific to 1st extracellular loop of human CD133 protein	Amino acid residues 303–312	WB, IHC, IF	Yes	Yes
D4W4N	Cell Signaling Technology	R/m	Recombinant protein corresponding to first extracellular domain of human CD133 protein	Amino acid residues 257–281, which include a single N-linked glycosylation site (Asn274)	WB, IHC	Yes	Yes
A3G6K	Cell Signaling Technology	R/m	Synthetic peptide corresponding to residues surrounding Asp562 of human CD133	Residues surrounding Asp562 of human CD133	WB	Yes	Yes
A8N6N	Cell Signaling Technology	M/m	Cells overexpressing human CD133 protein	No data	FC	No data	No data
MAB4399 (clone 17A6.1)	Merck Millipore (Burlington, MA, USA) Sigma-Aldrich	M/m	GST-tagged recombinant fragment corresponding to 77 amino acids from N-terminal region of human CD133	N-terminus	ICC, WB	No data	No data

* The list includes conventional and newly provided recombinant antibodies available at the moment of publication. Data are provided according to the manufacturers’ specifications; thus, the application potential in practice may be greater. Information about the immunogen and the ability to recognize glycosylated and non-glycosylated CD133 has been supplemented using information published in articles. Abbreviations: HCL—human cell line, CL—cell line, M—mouse, R—rabbit, m—monoclonal, FC—flow cytometry, MICS—MACSima Imaging Cyclic Staining, IF—immunofluorescence, IHC—immunohistochemistry, IHC-P—immunohistochemistry—paraffin, MC—mass cytometry, ICC—immunocytochemistry, WB—Western blotting, IP—immunoprecipitation, IHC-Fr—immunohistochemistry—frozen sections.

**Table 2:** Custom anti-human CD133 antibodies *.

Antibody Name/Clone	Host/Clonality	Immunogen	Recognized Epitope	Application	Recognition of Glycosylated CD133	Recognition of Non-Glycosylated CD133	Ref.
AC139	M/m	WERI-Rb-1 retinoblastoma cell line	No data	FC	No data	No data	[37]
AC140	M/m	WERI-Rb-1 retinoblastoma cell line	No data	FC	No data	No data	[37]
AC142	M/m	WERI-Rb-1 retinoblastoma cell line	No data	FC	No data	No data	[37]
αhE2	R/p	Gly240–Ser388	No data	IF, WB, IHC	Yes M = 105 kDa; M = 120 kDa	Yes M = 92 kDa	[97]
80B258	M/m	Gly240–Ser388 of second extracellular domain of human prominin-1	No data	IF, WB, IHC	Yes M = 105 kDa; M = 120 kDa	Yes M = 92 kDa	[27]
6B6	M/m	CD133-positive cell line Y79	No data	FC, IHC	No data	No data	[126]
Anti-D-EC3	M/p	Third domain (D-EC3) (serine 641–leucine 710) in *E. coli* BL21	Non-glycosylated fragment of EC3 loop	No data	No data	No data	[127]
HA10	HCL/Antibody phage display technique	Recombinant human CD133—native and deglycosylated	Glycosylation-independent epitope on extracellular domain 2 (EC2)	FC, IHC	No data	No data	[128]
C2E1	M/m	CD133 ectodomain 2 (amino acids 507–716)	CD133 ectodomain 2 (amino acids 507–716)	WB, IHC, ELISA	Yes M = 115 kDa	Yes M = 95 kDa	[129]
RW03-IgG	M/Antibody phage display technique	CD133-specific phage-displayed synthetic antibody fragment (Fab) from Library F	Unique epitope presented in glycosylated and non-glycosylated CD133	IF, FC	No data	No data	[130]
Anti-CD133	R/p	Synthetic peptide derived from N-terminal extracellular region of CD133 (amino acids 36–50)	N-terminal epitope of CD133	IF, ELISA	Yes M= 100–120 kDa	No data	[131]
CD133 47–10 (CPTC-*PROM1*-1)	R/m	Synthetic peptide amino acids 295–329	Domain comprising amino acids 180–400	IF, IHC, WB	Yes M = 120 kDa	Yes M = 93–97 kDa	[132]

* Data are provided according to the information published in articles. Abbreviations: HCL—human cell line, M—mouse, R—rabbit, m—monoclonal, FC—flow cytometry, IF—immunofluorescence, IHC—immunohistochemistry, WB—Western blotting, ELISA—enzyme-linked immunosorbent assay.
